# Home care for patients with COVID-19: Niger’s experience; about 2037 cases in the city of Niamey

**DOI:** 10.1016/j.ijregi.2026.100897

**Published:** 2026-04-15

**Authors:** Sahada Moussa Saley, Nassirou Saidou Chaibou, Boulama Malam Mamadou Boulama, Zaratou Ali, Oum Ramatou Ganda, Hanki Yahayé, Elisée Akoïna Abomtcho, Gado Mamadou Amadou, Ibrahim Alkassoum Salifou, Eric Adehossi

**Affiliations:** 1 Faculty of Health Sciences, Abdou Moumouni University, Niamey, Niger; 2 Infectious Diseases Department, Niamey National Hospital, Niamey, Niger; 3 Ministry of Public Health: COVID-19 Care Commission, Niamey, Niger

**Keywords:** COVID-19, Home monitoring, Niamey, Niger

## Abstract

**Objectives:**

This study aimed to describe the epidemiologic, clinical, therapeutic, and clinical course profiles of patients receiving home care for COVID-19 in the city of Niamey.

**Methods:**

This was a descriptive, retrospective, cross-sectional study conducted over a 1-year period, from April 2020 to March 2021, focusing on the management of patients with COVID-19 in Niamey. All patients presenting with a mild form of the disease, identified and monitored by mobile teams, were included in this study.

**Results:**

A total of 2037 patients were included. Men accounted for 69.1% (n = 1407). The mean age was 39 ± 15.1 years, ranging from 1 to 86 years. Screening of index cases accounted for 69.3% of diagnostic circumstances. Comorbidities were present in 24.4% of patients (n = 497), and 63.6% (n = 1295) were asymptomatic. Hydroxychloroquine and/or azithromycin were used in 98.7% of cases (n = 2010), and adverse effects related to hydroxychloroquine were observed in 1.0% (n = 19) of patients. During follow-up, 3.9% (n = 79) of patients were subsequently hospitalized. In total, 94.9% (n = 1932) were declared recovered, of whom 74.3% (n = 1432) between the 10th and 15th day of treatment. Finally, 0.7% (n = 15) of patients died, representing 19.0% of those who were subsequently hospitalized.

**Conclusion:**

The favorable outcome observed in the majority of patients demonstrates the effectiveness of home care for mild forms of COVID-19, enabling early detection of severe cases and rapid intervention. However, prevention remains the best way to combat this pandemic.

## Highlights

• Home care managed 2037 mild COVID-19 cases in Niamey, Niger.
• A total of 94.8% of patients recovered, most between days 10 and 15 of treatment.
• Hydroxychloroquine/azithromycin used in 98.6% of cases, with few side effects.
• Only 3.9% required hospitalization; the mortality rate was 0.7%.
• Home monitoring proved effective in resource-limited settings.

## Introduction

At the end of 2019, a new coronavirus, now known as SARS-CoV-2, was identified as the cause of a cluster of pneumonia cases in Wuhan, China. It spread rapidly, leading to a global pandemic, and was declared a Public Health Emergency of International Concern by the World Health Organization (WHO) on January 30, 2020 [1,2]. The WHO named the disease COVID-19: “CO” for corona, “VI” for virus, “D” for disease, and “19” for the year of its emergence [1]. Within a few weeks, the pandemic presented all countries with major challenges, particularly, low-income nations with fragile health systems or those already facing a health crisis.

Faced with the threat and severity of this health emergency, Niger, similar to many other countries, developed a national preparedness and response plan for COVID-19 [3].

The first case was reported on March 19, 2020. Fortunately, in Niger, mild forms of COVID-19 were the most common [4]. This situation proved advantageous because care could be provided at home due to the lack of adequate health care infrastructure, in accordance with the WHO’s interim guidelines updated on August 12, 2020 [5]. Home care is recommended for patients with confirmed COVID-19 when hospital care is unavailable or risky (e.g. due to a lack of health care resources), as well as for asymptomatic patients or those with mild symptoms. It may also be offered to patients after their discharge from hospital if necessary [3,5]. To support this strategy, the country has set up mobile home care teams.

It, therefore, seems essential to analyze the data from this monitoring to draw lessons and improve and adapt the response plan and patient care, hence the objective of our study.

## Patients and methods

### Study design

This was a descriptive cross-sectional study with retrospective recruitment, conducted over a 1-year period from April 2020 to March 2021 in the city of Niamey.

This study aimed to describe the clinical, paraclinical, therapeutic, and prognostic profile of patients receiving home care for mild SARS-CoV-2 infection.

### Study setting

**Figure 1:**
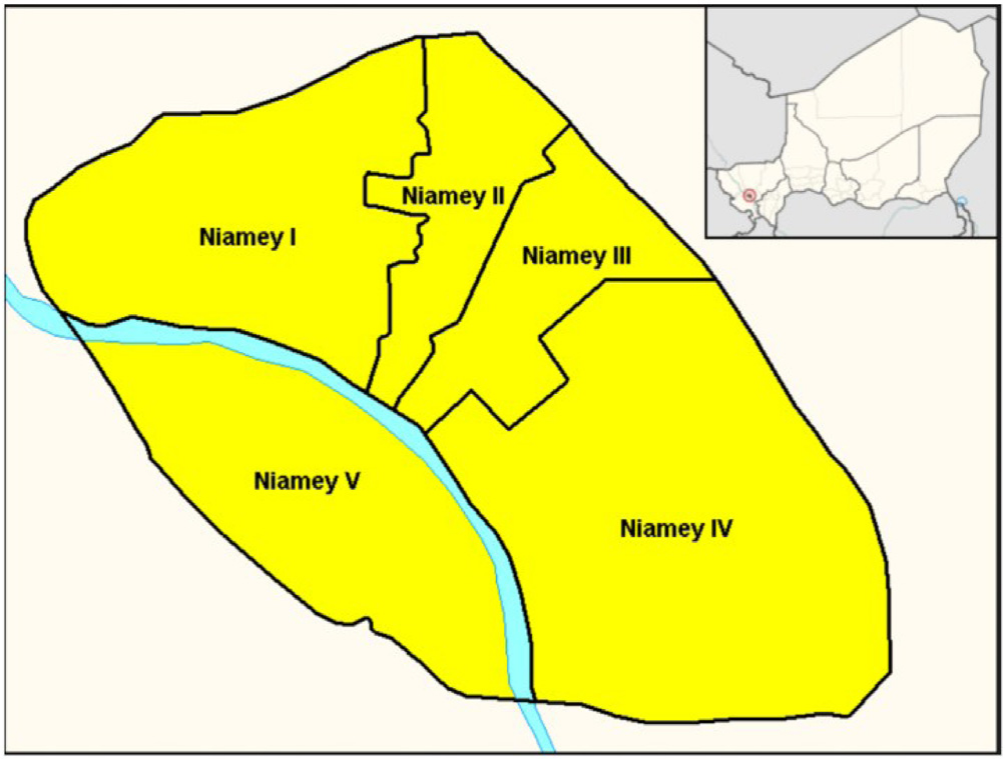
Map showing the five districts of Niamey. Figure 1 shows the distribution of the five districts of the city of Niamey. On the map of Niger, at the top right, the city of Niamey is marked by a red dot.

Niamey, the capital of Niger, is situated in the western part of the country. It covers an area of approximately 250 km² and is divided into five administrative districts (I-V). In 2026, its population was estimated at over 1.6 million inhabitants, predominantly young people, with a sex ratio close to 1. The city has the most well-structured health care network in the country. Its health care network consists mainly of national referral hospitals (Niamey National Hospital, Amirou Boubacar Diallo National Hospital, General Referral Hospital, Issaka Gazobi Maternity Hospital, National Tuberculosis Centre), two regional hospitals, and a number of Integrated Health Centres and private health centers. Figure 1 shows the layout of the five districts of the city of Niamey [[6], [7], [8], [9]].

District I of Niamey: It is situated on the right bank; this is the most urbanized area of the capital. It is home to numerous national and international institutions, as well as the residences of their staff whose international mobility is particularly high.

Its population was estimated at 314,043 in 2025, predominantly young people, with a sex ratio close to 1 [10].

Study population: the study included all patients with COVID-19 residing in Niamey who received home care and had a daily monitoring form correctly completed during the study period.

### Inclusion criteria

The study included patients aged under 70 years with a positive SARS-CoV-2 test (antigen [Ag] rapid test and/or polymerase chain reaction [PCR]) and meeting at least one of the following criteria:

▪ Asymptomatic patients, particularly, contact cases or travelers;
▪ Patients presenting with a mild form of the disease according to the WHO classification, without comorbidities or with stable comorbidities at the start of follow-up [3,5];
▪ Patients discharged from the hospital for COVID-19, clinically stable, followed up until the lifting of lockdown.

### Follow-up procedures

**Figure 2:**
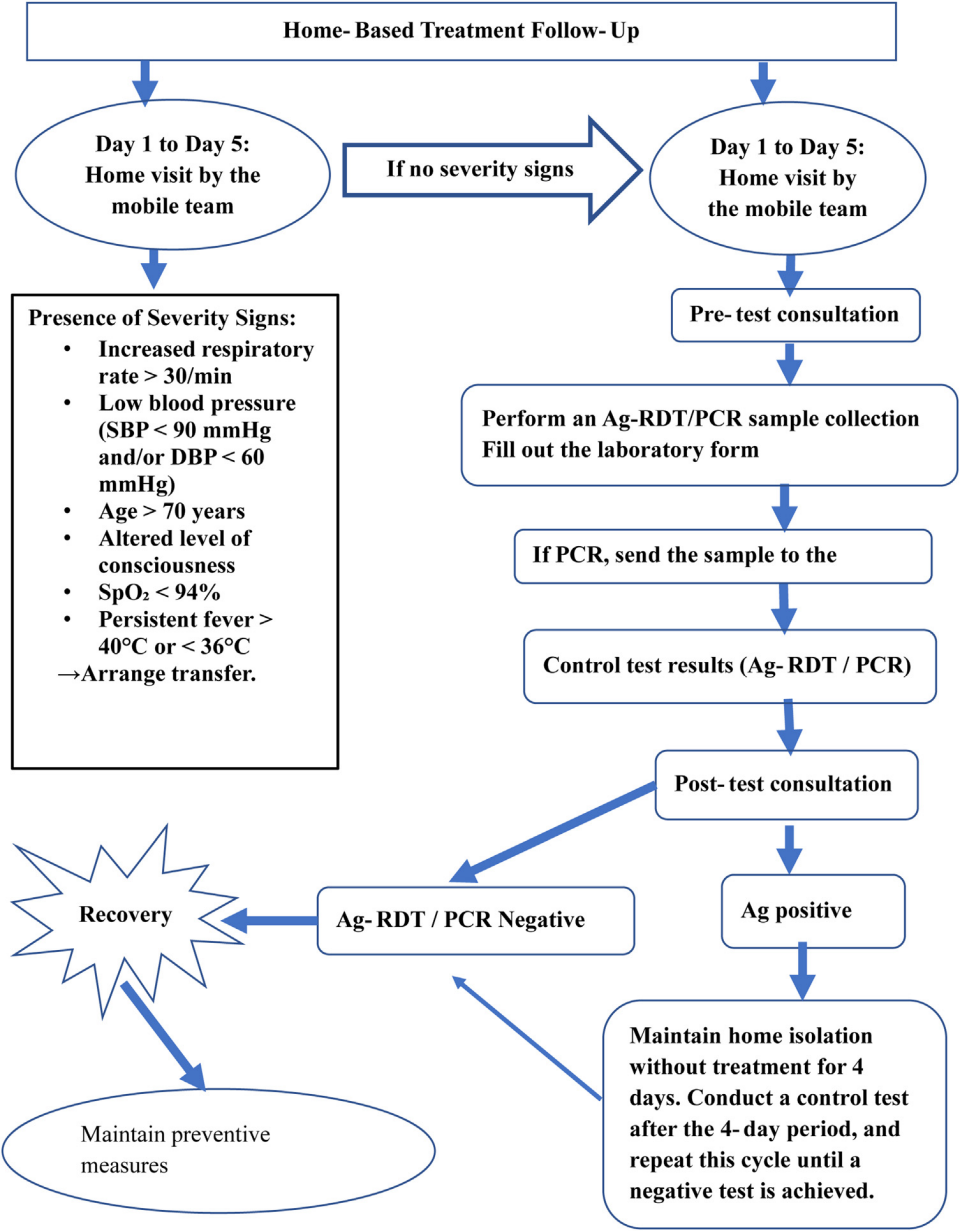
Home monitoring algorithm. Figure 2 details the process of home‑based follow‑up for patients.

This was carried out by designated health care staff trained for this purpose, under the coordination of a focal point. Monitoring involved home visits, carried out systematically from the first to the fifth day of follow-up, with visits as required and telephone calls continuing until the 10th day, followed by a confirmatory Ag rapid diagnostic test (RDT) or PCR test on the 11th day of treatment (if medication was prescribed) or the 11th day of follow-up. If the confirmatory Ag RDT or PCR test was negative, the patient was declared recovered and follow-up was concluded. Otherwise, follow-up continued with confirmatory Ag RDT or PCR tests every 4 days; throughout the follow-up period, signs of severity (see Figure 2) were actively monitored, and prompt hospitalization was arranged where necessary. Figure 2 presents the follow-up algorithm.

### Operational definitions

▪ Recovery was defined as a negative rapid diagnostic test (RDT) Ag and/or PCR test during home follow-up.
▪ A patient is considered lost to follow-up if they subsequently refuse follow-up or after three successive missed contacts.

### Variables studied

The variables collected included sociodemographic characteristics, circumstances of diagnosis, comorbidities, clinical signs, treatment received, and clinical progression.

### Data collection

Data were derived from home follow-up records completed daily by mobile teams. A consistency check was carried out before data entry.

### Statistical analysis

Data were analyzed using Epi Info version 7.2.2.6.

▪ Qualitative variables were presented as frequencies and proportions.
▪ Quantitative variables were expressed as mean and SD.

### Ethical considerations

This study was based on anonymized secondary data. Administrative authorization was obtained from the Governor of the Niamey region as part of a doctoral thesis (see administrative documents in the appendix). Patient confidentiality and anonymity were strictly respected.

## Results

### Sociodemographic characteristics

A total of 2037 patients were included, of whom 69.1% (n = 1407) were men, with a male-to-female ratio of 2.2. The mean age was 39 ± 15.1 years, ranging from 1 to 86 years. The 31-40 age group accounted for 23.7% (n = 483) of the patients (Table 1). Most patients (40.20%) were from Commune I of Niamey.

**Table 1:** Distribution of patients by age group.

Age range (year)	Frequency	Percentage (%)
**1-10**	**68**	**3.3**
**11-20**	**148**	**7.3**
**21-30**	**383**	**18.8**
**31-40**	**483**	**23.7**
**41-50**	**468**	**23.0**
**51-60**	**300**	**14.7**
**61-70**	**131**	**6.4**
**71-80**	**40**	2.0
**>80**	**16**	**0.8**
**Total**	**2037**	**100.0**

The distribution of patients by age group shows a predominance of young adults, particularly, those aged 31-40 years (23.7%) and 41-50 years (22.9%). The youthfulness of the studied population may explain the mild nature of COVID-19 in this group.

### Clinical aspects

**Figure 3:**
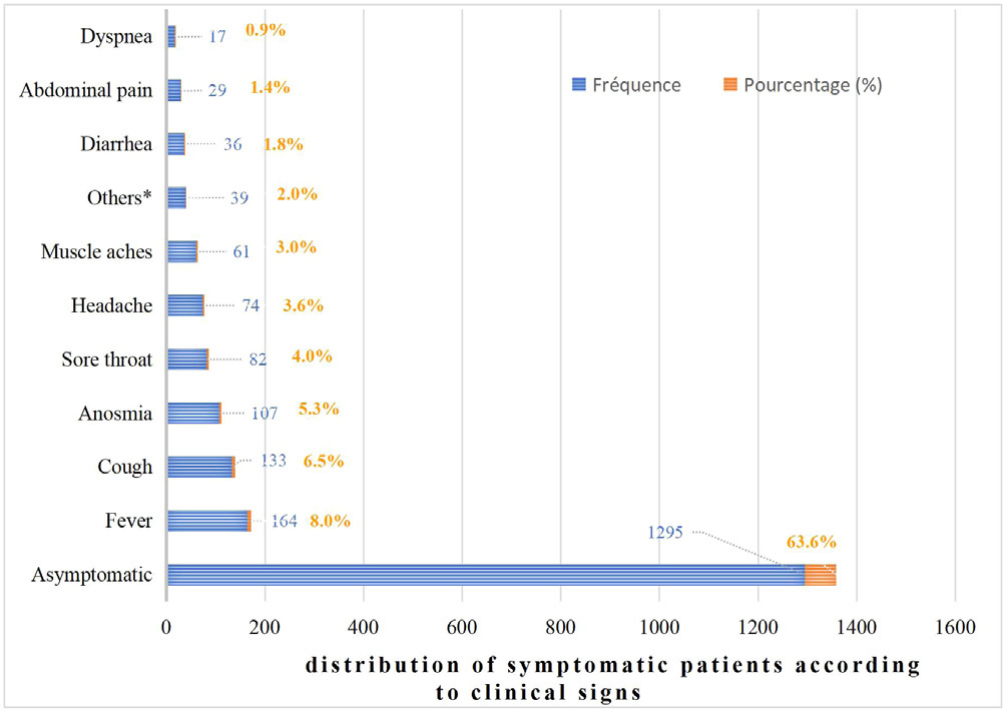
Distribution of symptomatic patients according to clinical gaps. Figure 3 shows the distribution of patients according to the clinical signs observed. It is noteworthy that the majority of cases (63.6%) were asymptomatic.

In this study, 24.4% (n = 497) of the patients had comorbidities, the most common being hypertension (11.0%). Furthermore, 69.3% (n = 1411) of the patients were identified during screening (Table 2). Asymptomatic cases accounted for 63.6% (n = 1295), whereas 8% (n = 164) presented with fever (Figure 3).

**Table 2:** Distribution of patients according to comorbidities and circumstances of diagnosis.

Distribution of patients according to their comorbidities		
Comorbidities	Frequencies	Percentages (%)
**Without comorbidities**	**1540**	**75.6**
**High blood pressure**	**225**	**11.1**
**Diabetes**	**111**	**5.5**
**Asthma**	**91**	**4.5**
**Others**	**29**	**1.4**
**Sickle cell disease**	**26**	**1.3**
**Tobacco**	**15**	**0.7**
**Total**	**2037**	**100.0**

Other: stroke, heart failure, sinusitis, pneumonia, obesity		
Distribution of patients according to the circumstances of diagnosis		
Circumstance of detection	Frequencies	Percentages (%)
**Contact of a positive case**	**1411**	**69.3**
**Departure traveler test**	**433**	**21.3**
**Suspicious**	**98**	**4.8**
**Not specified**	**60**	**3.0**
**Arrival traveler test**	**35**	**1.7**
**Total**	**2037**	**100.0**

This table illustrates the distribution of patients according to their comorbidities and the circumstances of diagnosis. The majority had no comorbidities, and detection occurred mainly among contacts of positive cases.

Among the patients, 98.7% (n = 2010) accepted treatment (hydroxychloroquine and/or azithromycin) and the majority (95.5%) received it within 3 days of diagnosis, with 85.3% being treated with hydroxychloroquine and 1% (n = 19) reporting adverse effects related to it, palpitations being the most frequently reported (33.1% of adverse effects).

**Figure 4:**
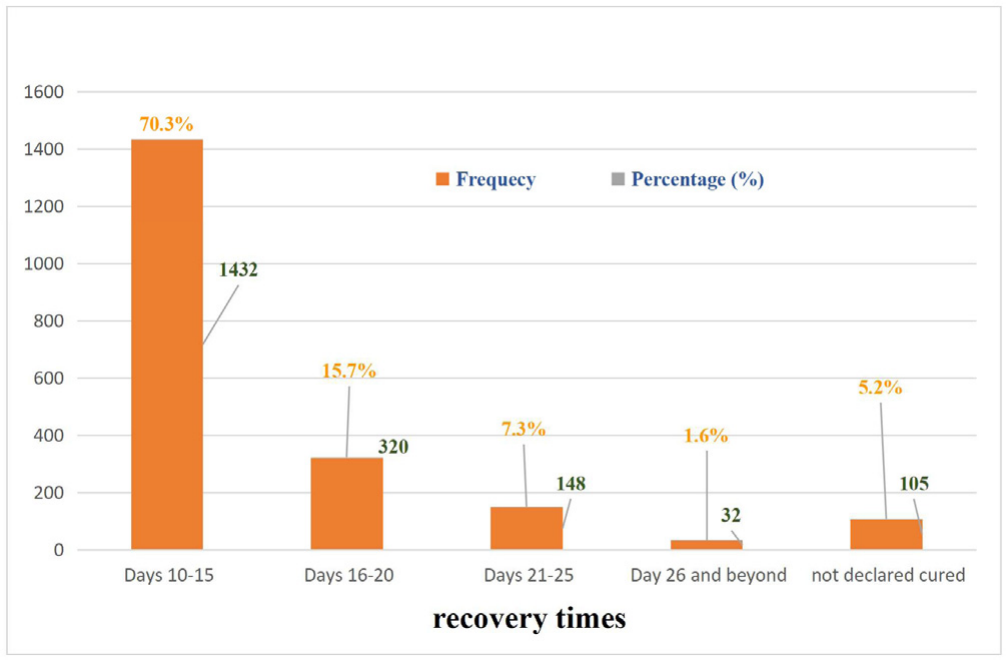
Recovery times. Figure 4 shows the distribution of patients according to their recovery time. The majority (70.3%) recovered within 10-15 days.

During follow-up, 4.4% of the patients (n = 90) were lost to follow-up and 3.9% (n = 79) were subsequently hospitalized. All patients who agreed to follow-up tests were declared cured, representing 94.9% of the patients (n = 1932), of whom 74.3% (1432) were declared cured between the 10th and 15th day of treatment (Figure 4). In our cohort, the mortality rate was 0.7% (n = 15), representing 19.0% of the patients subsequently hospitalized (Table 3).

**Table 3:** Outcome of the monitoring.

Secondary hospitalization or not			
	Frequency	Percentage (%)	
**Subsequently hospitalized**	**79**	**3.9**	
**Non-hospitalized patients**	**1958**	**96.1**	
**Total**	**2037**	**100.0**	

Evolution/prediction			
		Frequency	Percentage (%)
**Declared cured** **n = 1932 (94.85%)**	**Only at home**	**1868**	**91.7**
	**They were subsequently hospitalized**	**64**	**3.1**
**Lost sight of**	**Secondarily hospitalized patients**	0	00.0
	**Residence**	**90**	**4.4**
**Deceased**	**They were subsequently hospitalized**	**15**	**0.7**
**Total**		**2037**	**100.0**

The vast majority of patients (96.1%) did not require secondary hospitalization. Overall, 94.9% were declared cured, most of them being followed up exclusively at home. Subsequent hospitalizations and deaths remained rare, confirming the predominance of mild forms in this cohort.

## Discussion

Our study included 2037 patients, 69.1% of whom were men (male-to-female ratio of 2.2). Several authors have also reported this male predominance [[11], [12], [13], [14], [15], [16]], possibly because men are more active and, therefore, more exposed (Figure 3, Figure 4).

The mean age of the patients was 39 years, similar to that of the study conducted in 2021 by Camara *et al.* [17] in Mali (40 years) but younger than the results obtained by Guan *et al.* [18] in China (47 years), by Gautret *et al.* [19] in Marseille (52 years), by Chaytee *et al.* [15] in Paris (51 years), and by Bouinoune *et al.* [20] in Martinique (45 years). This may be explained by the fact that the African population is younger. Furthermore, unlike the majority of these studies, our work also included asymptomatic patients.

The majority of our patients (69.3%) were identified through screening tests. This result is higher than that of the study by Coulibaly *et al.* [21] conducted in 2021 in Mali (45.0%). Indeed, Coulibaly *et al.* [21]’s study was conducted in a hospital setting where diagnoses were made only in sick individuals, whereas our study also included asymptomatic patients identified through screening and mildly symptomatic patients who were not hospitalized.

In our study, 75.6% of the patients had no comorbidities and 63.6% were asymptomatic. These findings may be explained by the fact that our cohort was generally younger. Older people are more likely to have comorbidities, present with symptoms, or develop severe forms of COVID-19, which often require hospitalization. A study conducted by Amankaye [12] in Niamey among hospitalized patients revealed a median age of 60 years and a higher rate of comorbidities (43%), with a significant association between age group and comorbidities (*P* <0.05) [14].

Almost all patients agreed to treatment, and most (95.5%) received it within 3 days of diagnosis, with 85.3% of them being treated with hydroxychloroquine. According to an international survey conducted in March 2020, 33% of doctors reported prescribing (or seeing their colleagues prescribe) hydroxychloroquine or chloroquine, and 41% reported using azithromycin or similar antibiotics. Among those who had treated patients with COVID-19, 37% considered hydroxychloroquine to be the most effective treatment, and 32.0% felt the same about azithromycin [22].

The toxicity of these two drugs is not a major concern [19]. Only 1.0% of our patients experienced adverse effects related to hydroxychloroquine, the most common being palpitations (33.1%). These effects led to hydroxychloroquine being replaced with azithromycin.

Among our patients, 91.7% made a favorable recovery at home and only 3.9% required readmission to hospital. This rate is lower than that reported in France, notably, by Chaytee *et al.* [15] in Paris, Lejeune *et al.* [16] in Montpellier, and Bouinoune *et al.* [20] in Martinique, who reported secondary hospitalizations of 9.3%, 16.1%, and 8.1%, respectively. In our cohort, patients were younger, had fewer comorbidities, and developed milder forms of COVID-19, which could explain this difference, particularly, compared with Chaytee *et al.* [15]’s cohort consisting of patients initially hospitalized and then discharged while on oxygen therapy. Also, this follow-up very likely helped prevent the overload of hospital staff and enabled the early detection of patients requiring secondary hospitalization. Finally, it opens the way for a broader reflection on the role played by such follow-up in the management of COVID-19.

Among our patients reported as recovered, 74.3% had recovered between the 10th and 15th day. A Chinese study reported a median duration of viral shedding of 20 days (interquartile range 17-24), with extremes ranging from 8 to 37 days [23]. This confirms the mild nature of most cases in our series and the effectiveness of home management.

In our cohort, the mortality rate was 0.7%. This rate is lower than that reported in the study by Chaytee *et al.* [15] in Paris, which found a mortality rate of 1.0%. This difference is mainly attributable to the young age of our patients, the relative rarity of comorbidities, and the benign presentation at the start of follow-up in our study.

Among patients requiring subsequent hospitalization, the mortality rate was 19%, lower than the 35.5% reported by Amankaye [12] in Niamey in 2022. This highlights the valuable contribution of home-based care. In general, decisions regarding referral depend on the patient’s assessment and are usually made at more advanced stages of the disease. Home-based care has likely contributed to earlier identification of the need for consultation and hospitalization, enabling rapid management, thereby reducing morbidity and mortality.

## Conclusion

COVID-19, an emerging infectious disease caused by the SARS-CoV-2 virus that first appeared in China, has become a global public health issue that has taken the world by surprise. Overall, this study shows that the course of COVID-19 is often mild in our setting, making home care a viable option. The favorable outcomes observed in our patients and the lower mortality rates demonstrate the effectiveness of home care for mild forms of COVID-19, enabling early detection of severe cases and prompt treatment. This also helps to avoid the constraints and complications associated with hospitalization. Nevertheless, prevention remains the best strategy for combating this pandemic.

## Declaration of competing interest

The authors have no competing interests to declare.

## Appendix Supplementary materials

Image, application 1
