# Supplementary figures and images for: Home care for patients with COVID-19: Niger’s experience; about 2037 cases in the city of Niamey
Source: IJID Reg. 2026 Apr 15;19:100897. doi: 10.1016/j.ijregi.2026.100897 (PMC13196440; doi:10.1016/j.ijregi.2026.100897)

**ANNEXES**

**
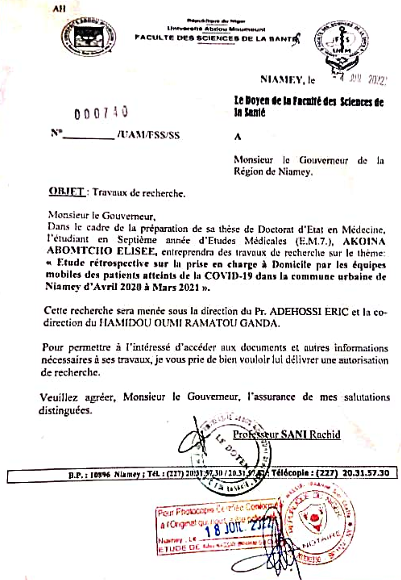
ANNEX 1**

**
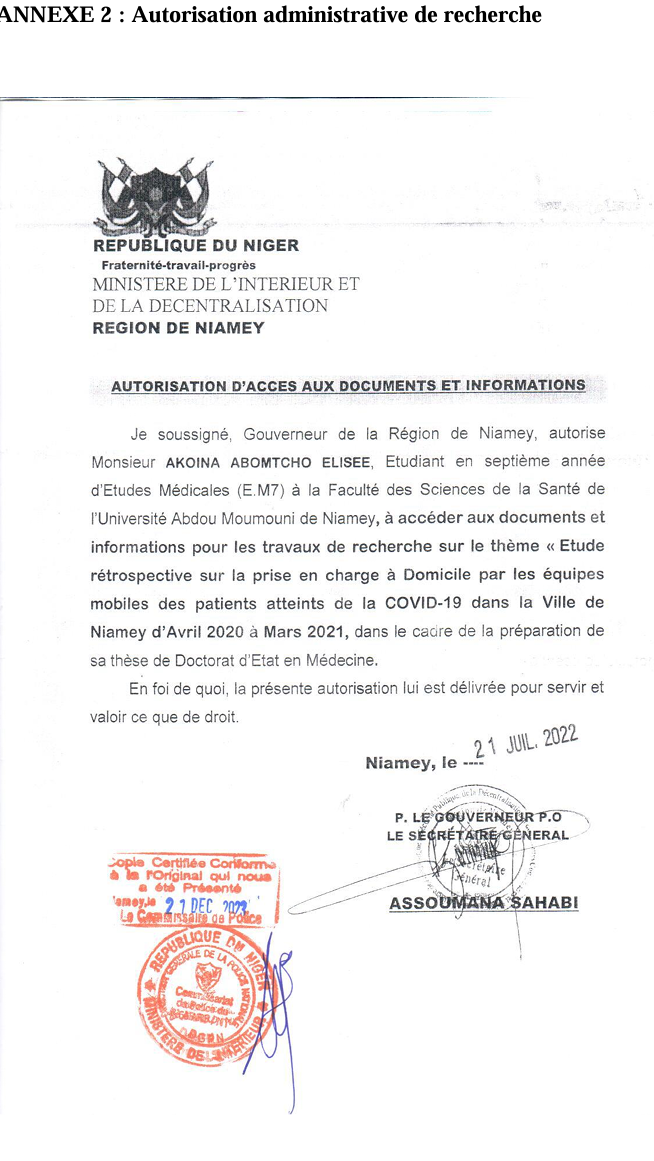
**

Supplement: Supplementary file 1 [file mmc1.docx]
